# Empirically characteristic analysis of chaotic PID controlling particle swarm optimization

**DOI:** 10.1371/journal.pone.0176359

**Published:** 2017-05-04

**Authors:** Danping Yan, Yongzhong Lu, Min Zhou, Shiping Chen, David Levy

**Affiliations:** 1 College of Public Administration, Huazhong University of Science and Technology, Wuhan, Hubei, China; 2 Non-traditional Security Center of Huazhong University of Science and Technology, Wuhan, Hubei, China; 3 School of Software Engineering, Huazhong University of Science and Technology, Wuhan, Hubei, China; 4 Data61, Commonwealth Scientific and Industrial Research Organization, Marsfield, New South Wales, Australia; 5 School of Electrical and Information Engineering, University of Sydney, Sydney, New South Wales, Australia; Beihang University, CHINA

## Abstract

Since chaos systems generally have the intrinsic properties of sensitivity to initial conditions, topological mixing and density of periodic orbits, they may tactfully use the chaotic ergodic orbits to achieve the global optimum or their better approximation to given cost functions with high probability. During the past decade, they have increasingly received much attention from academic community and industry society throughout the world. To improve the performance of particle swarm optimization (PSO), we herein propose a chaotic proportional integral derivative (PID) controlling PSO algorithm by the hybridization of chaotic logistic dynamics and hierarchical inertia weight. The hierarchical inertia weight coefficients are determined in accordance with the present fitness values of the local best positions so as to adaptively expand the particles’ search space. Moreover, the chaotic logistic map is not only used in the substitution of the two random parameters affecting the convergence behavior, but also used in the chaotic local search for the global best position so as to easily avoid the particles’ premature behaviors via the whole search space. Thereafter, the convergent analysis of chaotic PID controlling PSO is under deep investigation. Empirical simulation results demonstrate that compared with other several chaotic PSO algorithms like chaotic PSO with the logistic map, chaotic PSO with the tent map and chaotic catfish PSO with the logistic map, chaotic PID controlling PSO exhibits much better search efficiency and quality when solving the optimization problems. Additionally, the parameter estimation of a nonlinear dynamic system also further clarifies its superiority to chaotic catfish PSO, genetic algorithm (GA) and PSO.

## 1 Introduction

The emergence of chaotic systems was initially described by Lorenz [1] and by Hénon [2]. The two famous chaotic attractors bearing their names are the cornerstone of chaos theory in modern literatures. Chaos can be described as a deterministic behaviorial characteristic of bounded nonlinear systems. Chaotic systems generally exhibit the following properties: sensitive to initial conditions, topologically mixing, and dense in periodic orbits. Although they usually appear to be stochastic, they are conditionally deterministic and periodically ergodic through the whole search space. These distinct merits have caused great concerns from many scientific disciplines including geology, mathematics, microbiology, biology, computer science, economics, engineering, finance, algorithmic trading, meteorology, philosophy, physics, politics, population dynamics, psychology, and robotics. Up to now, chaos theory has become a very active area of research and its applicability is also vastly broadened. Scholars and practitioners all over the world make full use of it to investigate the control, synchronization, prediction and optimization problems of nonlinear dynamic systems by following chaotic ergodic orbits.

As is known, finding out optimal solutions is a hard and significant task in a good many nonlinear dynamic systems. Optimization problem solving is chiefly concerned about the quantitative and qualitative study of optima to pursue and the methods of finding out them. The emergent optimization techniques are usually divided into three distinct classes: natural phenomena, physical phenomena and mathematical computational phenomena. They often tend to exploit evolutionary heuristics to solve the solutions. In addition, being deterministic and ergodic, chaos is combined with evolutionary heuristics and acts as a prominent role in solving optimization problems. There exist two chaotic ways to be applied to optimization areas [3–5]. The first way is to introduce chaos into a unified ensemble like neural network. The harmonic combination of neurons and non-equilibrium dynamics with diverse concomitant attractors can completely use chaotic ergodic orbits to pursue the global optimum. The another way is to closely combine evolutionary variables with chaotic attractors and edges. Their generic philosophy is as follows: mapping the relevant variables or ensemble in the problems from the chaotic space to the search space, and then utilizing chaotic ergodic orbits to search the optima instead of using random orbits. Meanwhile, in order to obtain the objective, sensitivity to initial conditions has to be taken into consideration seriously. More inspiringly, great progresses pertaining to chaotic optimization heuristics have been made in the past decade [6–14]. Simultaneously, some recent remarkable work on the study of PSO is worth noting [15–18].

Liu et al. proposed a hybrid particle swarm optimization algorithm by incorporating logistic chaos and adaptive inertia weight factor into PSO, which reasonably combines the population-based evolutionary PSO search ability with chaotic search behavior [6]. In [7], Cai et al. presented a chaotic PSO method based on the tent equation to solve economic dispatch problems with generator constraints. Compared with the traditional PSO method, the chaotic PSO method has good convergence property accompanied by the lower generation costs and can result in great economic effect. Hong elucidated the feasibility of applying a chaotic PSO algorithm to choose the suitable parameter combination for a support vector regression model. The optimized model provides the theoretical exploration of the electric load forecasting support system [8]. In [9], Wang and Liu proposed a logistic chaotic PSO approach to generate the optimal or near-optimal assembly sequences of products. The proposed method is validated with an illustrative example and the results are compared with those obtained using the traditional PSO algorithm under the same assembly process constraints. Chuang et al. presented accelerated chaotic PSO with an acceleration strategy and used it to search through arbitrary data sets for appropriate cluster centers. Results of the robust performance from accelerated chaotic PSO indicate that this method an ideal alternative for solving data clustering problem [10]. In [11], Chuang et al. proposed chaotic catfish PSO. Statistical analysis of the experimental results indicate that the performance of chaotic catfish PSO is better than the performance of PSO, chaotic PSO, catfish PSO. In [12], Wang et al. developed an approach for grey forecasting model, which is particularly suitable for small sample forecasting, based on chaotic PSO and optimal input subset. The numerical simulation result of financial revenue demonstrates that developed algorithm provides very remarkable results compared to traditional grey forecasting model for small dataset forecasting. More recently, Gandomi et al. introduced chaotic accelerated PSO and applied it to solving three engineering problems. The results show that chaotic accelerated PSO with an appropriate chaotic map can clearly outperform standard accelerated PSO, with very good performance in comparison with other algorithms and in application to a complex problem [13]. In [14], Xu et al. presented a novel robust hybrid PSO based on piecewise linear chaotic map and sequential quadratic programming. This novel algorithm makes the best of ergodicity of the piecewise linear chaotic map to help PSO with the global search while employing the sequential quadratic programming to accelerate the local search. Qin et al. presented an improved PSO algorithm with an interswarm interactive learning strategy by overcoming the drawbacks of the canonical PSO algorithm’s learning strategy. The algorithm is inspired by the phenomenon in human society that the interactive learning behavior takes place among different groups [15]. Zhang et al. proposed a novel vector coevolving particle swarm optimization algorithm [16]. Du et al. presented a heterogeneous strategy PSO, in which a proportion of particles adopts a fully informed strategy to enhance the converging speed while the rest is singly informed to maintain the diversity [17]. Niu et al. proposed a new variant of PSO, named symbiosis-based alternative learning multiswarm particle swarm optimization [18].

Since PID controllers can successfully adopt a weighted PID term sum to determine a new control variable and further minimize the error over time between a desired setpoint variable and a measured process variable, PID controlling law has been widely used in various industry control systems. Since Lu et al. proposed a PID controlling PSO algorithm and successfully applied it to estimating the parameters of vertical takeoff and landing aircrafts [19, 20]. Therefore, in this paper, in order to improve the performance of the algorithm and broaden its more applications, we propose a novel hybrid PSO algorithm which we call chaotic PID controlling PSO. The hierarchical inertia weight coefficients, PID controller, and chaotic logistic map are simultaneously incorporated into PSO to improve the PSO nonlinear dynamics. The hierarchical inertia weight coefficients are determined in accordance with the present fitness values of the local best positions. The chaotic logistic map is used in both the substitution of the two random parameters and the chaotic local search of for the global best position. Successively, the convergent analysis of chaotic PID controlling PSO is deeply investigated. For the purpose of performance evaluation of chaotic PID controlling PSO, empirical experiments are conducted on some complex multimodal functions. Then it is further used in estimating the parameters of a nonlinear dynamic system in engineering. These simulation results prove the better effectiveness and efficiency of chaotic PID controlling PSO when solving the optimization problems, compared with other chaotic PSO algorithms and meta-heuristics such as chaotic PSO with the logistic map [6], chaotic PSO with the tent map [7], chaotic PSO [10], chaotic catfish PSO [11], pure random search (PRS) [21], GA [22], multistart (MS) [23], simulated annealing (SA) [23], taboo search (TS) [24], standard PSO (SPSO) [25, 26], chaotic simulated annealing (CSA) [27] and center PSO (CenterPSO) [28].

The remainder of the paper is organized as follows. Section 2 depicts the dynamical model, hierarchical inertia weight, chaotic local search for the global best position, whole procedure and convergent analysis of chaotic PID controlling PSO. Section 3 presents the experimental study of conducting chaotic PID controlling PSO on some complex multimodal functions together with other chaotic PSO in [6, 7, 11]. Section 4 depicts the application of parameter estimation of a nonlinear dynamic system using chaotic PID controlling PSO. Section 5 gives the conclusions and future work.

## 2 Analysis and methods

### 2.1 Representation of chaotic PID controlling PSO

SPSO is a stochastic population-based algorithm which is modeled on the behaviors of insects swarming, animals herding, birds flocking, and fish schooling where these swarms search for food in a collaborative manner, and it was originally introduced by Kennedy and Eberhart in 1995 [25, 26]. It is usually used for the optimization of continuous nonlinear systems. Since SPSO uses a simple swarm emulating mechanism to guide the particles to search for globally optimal solutions and implements easily, it has succeed in solving many real-world optimization problems. In order to improve the performance of the SPSO algorithm and achieve the specific goals of accelerating convergence speed and avoiding local optima, we herein bring forward a novel PSO approach called CPIDSO.

In this part, we discuss the dynamical model, hierarchical inertia weight, chaotic local search for the global best position, and give a full description of the procedure of chaotic PID controlling PSO in turn.

#### 2.1.1 Dynamical model of chaotic PID controlling PSO

SPSO is a kind of typically stochastic standard algorithm to search for the best solution by simulating the movement of the flocking of birds or fish. It works by initializing a flock of birds or fish randomly over the searching space, where each bird or fish is called a particle. These particles fly with certain velocities and find the global best position after some generations. At each generation, they are dependent on their own momentum and the influence of their own local and global best positions *x*_*lbest*_ and *x*_*gbest*_ to adjust their own next velocity *v* and position *x* to move in turn. SPSO is clearly depicted as follows

$$\begin{array}{c} v\left(t+1\right)=\omega_{pso}\cdot v\left(t\right)+c_{1}\cdot rand_{1}\cdot \left(x_{lbest}-x\left(t\right)\right)+c_{2}\cdot rand_{2}\cdot \left(x_{gbest}-x\left(t\right)\right) \end{array}$$ (1)

$$\begin{array}{c} x(t+1)=v(t+1)+x(t) \end{array}$$ (2),

where *ω*_*pso*_, *c*_1_ and *c*_2_ denote the inertia weight coefficient, cognitive coefficient and social coefficient, respectively, and *rand*_1_, *rand*_2_ are both random values between 0 and 1. Besides, *v* is clamped to a given range [-*v*_*max*_, + *v*_*max*_].

Supposing *ϕ*_1_ = *c*_1_ ⋅ *rand*_1_, *ϕ*_2_ = *c*_2_ ⋅ *rand*_2_, *ϕ* = *c*_1_ ⋅ *rand*_1_+*c*_2_ ⋅ *rand*_2_ and $\theta =\frac{\phi_{2}}{\phi }$, after introducing a proper PID controller into Eqs (1) and (2) [19, 20], we may obtain the following Eq (3). Please note that *t* in the Eq (3) denotes the present iterative generation and are not the absolute time metric. Actually, the PSO system is a continuous system. Therefore, we have used the PID controlling model in the context.

$$\begin{array}{c} \begin{array}{cc} v(t+1)= & w_{pso}\cdot v\left(t\right)\\ & +\phi \cdot (\left(1-\theta \right)\cdot \left(k_{p}\cdot \left(x_{lbest}-x\left(t\right)\right)+k_{i}\cdot \int_{0}^{t}\left(x_{lbest}-x\left(t\right)\right)\cdot dt+k_{d}\cdot \frac{d(x_{lbest}-x\left(t\right))}{dt}\right)\\ & +\theta \cdot (k_{p}\cdot \left(x_{gbest}-x\left(t\right)\right)+k_{i}\cdot \int_{0}^{t}\left(x_{gbest}-x\left(t\right)\right)\cdot dt+k_{d}\cdot \frac{d(x_{gbest}-x\left(t\right))}{dt})) \end{array} \end{array}$$ (3),

where $k_{p}=e^{\left(w_{pso}-1\right)\cdot \frac{t}{MaxT}}$, $k_{i}=\frac{e^{\left(w_{pso}-1\right)\cdot \frac{t}{MaxT}}}{1+e^{\left(w_{pso}-1\right)\cdot \frac{t}{MaxT}}}$, $k_{d}={\left[e^{\left(w_{pso}-1\right)\cdot \frac{t}{MaxT}}\right]}^{2}$, *MaxT* is the maximum generation.

If the random parameters *rand*_1_ and *rand*_2_ in Eq (1) of SPSO are chaotic, they can ensure the optimal ergodicity throughout the search space. Furthermore, there are no fixed points, periodic orbits, or quasi-periodic orbits in the behaviors of the chaotic systems. Therefore, they are necessarily substituted by the two sequences *Cr*^(*t*)^ and (1 − *Cr*^(*t*)^) generated via the following logistic map Eq (4)

$$\begin{array}{c} Cr^{\left(t+1\right)}=\mu \cdot Cr^{\left(t\right)}\cdot \left(1-Cr^{\left(t\right)}\right),i=0,1,2,\cdots ,n. \end{array}$$ (4),

where *Cr*^(0)^ is generated randomly for each independent run, but it is not equal to {0, 0.25, 0.5, 0.75, 1}, and *μ* is equal to 4. Obviously, *Cr*^(*t*)^ is distributed in the interval (0, 1.0). So the driving parameter *μ* of the logistic map controls the behavior of *Cr*^*t*^ as the iteration number *t* goes to infinity. So *ϕ* and *θ* are changed into the following Eqs (5) and (6).

$$\begin{array}{c} \phi =c_{1}\cdot Cr^{\left(t\right)}+c_{2}\cdot \left(1-Cr^{\left(t\right)}\right) \end{array}$$ (5)

$$\begin{array}{c} \theta =\frac{c_{2}\cdot \left(1-Cr^{\left(t\right)}\right)}{c_{1}\cdot Cr^{\left(t\right)}+c_{2}\cdot \left(1-Cr^{\left(t\right)}\right)} \end{array}$$ (6)

Concerning the inertia weight coefficient, we adopt the following hierarchical Eq (7) [6]

$$\begin{array}{c} w_{pso}=\left\{\begin{array}{c} \begin{array}{cc} {w_{pso}}_{min}+\frac{\left({w_{pso}}_{max}-{w_{pso}}_{min}\right)\left(f-f_{min}\right)}{f_{avg}-f_{min}}, & f\leq f_{avg}\\ {w_{pso}}_{max}, & f>f_{avg} \end{array} \end{array}\right. \end{array}$$ (7),

where $w_{Pso_{max}}$ and $w_{Pso_{min}}$ represent the maximum and minimum of *w*_*pso*_, *f* is the current objective value of the particle, *f*_*avg*_ and *f*_*min*_ are the average and minimum objective values of all particles, respectively. In addition, the cognitive coefficient is supposed to decrease linearly from 2 to 0 while the social coefficient is supposed to increase linearly from 0 to 2.

Consequently, our proposed chaotic PID controlling PSO is comprised of Eqs (2) and (3).

#### 2.1.2 Chaotic local search of chaotic PID controlling PSO

In chaotic PID controlling PSO, we introduce the following logistic Eq (8) in the process of the chaotic local search for the global best position *x*_*gbest*_ to improve the mutation mechanism

$$\begin{array}{c} C{x_{gbest,i}}^{\left(t+1\right)}=\mu \cdot C{x_{gbest,i}}^{\left(t\right)}\cdot \left(1-C{x_{gbest,i}}^{\left(t\right)}\right),i=1,2,\cdots ,n \end{array}$$ (8),

where *Cx_gbest,i_*^(*t*)^ denotes the *ith* chaotic variable, and *μ* is equal to 4. Obviously, *Cx_gbest,i_*^(*t*)^ is distributed in the interval (0, 1.0) under the conditions that the initial *Cx_gbest,i_*^(0)^ ∈ (0, 1) and that *Cx_gbest,i_*^(*0*)^ ∉ {0.25, 0.5, 0.75}. In general, the chaotic variable has special properties of ergodicity, pseudo-randomness and irregularity. Since a minute difference in the initial value of the chaotic variable would result in a considerable difference in its long time behavior, the chaotic variable can travel ergodically over the whole search space. Therefore, these merits of the chaotic variable can help the global optimum keep away from the local optima.

The procedure of the chaotic local search for the global best position based on the above-mentioned logistic Eq (8) can be illustrated as follows:

Step 1: Set *t* = 0 and map the decision variables *x_gbest,i_*^(*t*)^ *i* = 1, 2, …, *n* among the intervals (*x*_*min*, *i*_, *x*_*max*, *i*_) to the chaotic variables *Cx_gbest,i_*^(*t*)^ located in the intervals (0, 1) using the following Eq (9).

$$\begin{array}{c} C{x_{gbest,i}}^{\left(t\right)}=\frac{x_{gbest,i}^{\left(t\right)}-x_{min,i}}{x_{max,i}-x_{min,i}},i=1,2,\cdots ,n \end{array}$$ (9)

Step 2: Determine the chaotic variables *Cx_gbest,i_*^(*t* + 1)^ for the next iteration using the logistic Eq (8) according to *Cx_gbest,i_*^(*t*)^.

Step 3: Convert the chaotic variables *Cx_gbest,i_*^(*t* + 1)^ to the decision variables *x_gbest,i_*^(*t* + 1)^ using the following Eq (10).

$$\begin{array}{c} {x_{gbest,i}}^{\left(t+1\right)}=x_{min,i}+C{x_{gbest,i}}^{\left(t+1\right)}\left(x_{max,i}-x_{min,i}\right),i=1,2,\cdots ,n \end{array}$$ (10)

Step 4: Evaluate the new solution with the decision variables *x_gbest,i_*^(*t* + 1)^.

Step 5: If the new solution is better than the predefined criterion or the predefined maximum iteration reaches output the new solution as the result of the chaotic local search for the global position; otherwise, let *t* = *t* + 1 and go back to Step 2.

#### 2.1.3 Procedure of chaotic PID controlling PSO

Consequently, based on the aforementioned contexts, our proposed chaotic PID controlling PSO can be depicted below in detail.

Step 1: Initialize parameters including the number *PN* of particles, dimensional size *D* of each particle, maximum generation number *MaxT*, initial chaotic logistic values *Cr*^(0)^ and *Cx*^(0)^, initial chaotic tent value *Cx*1^(0)^, initial position *x* and velocity *v* of each particle, inertia weight coefficient *w*_*pso*_, and cognitive coefficient *c*_1_, social coefficient *c*_2_. Calculate the initial fitness of each particle, and set the initial local best position *x*_*lbest*_ and global best position *x*_*gbest*_.

Step 2: Calculate the three parameters *k*_*p*_, *k*_*i*_ *and* *k*_*d*_ of the PID controller. Then in terms of Eqs (2) and (3), calculate the next velocity *v*(*t*) and position *x*(*t*) of each particle. Next, calculate the fitness of each particle, set the local best position *x*_*lbest*_ and the global best position *x*_*gbest*_.

Step 3: If the fitness of the global best position is the same value seven times, then implement the chaotic local search for the global best position, and update the global best position using the result of Eq 10.

Step 4: Observe if the global best fitness(*x*_*gbest*_) meets the given stopping threshold or not, or observe if the maximum generation number *MaxT* reaches or not. If not, go back to Step 2.

Step 5: Otherwise, the operation can be terminated. Finally, output the global best position *x*_*gbest*_, and its corresponding global best fitness as well as convergent generation number.

The pseudo-code for chaotic PID controlling PSO is presented below in Algorithm 1.

**Algorithm 1** Chaotic PID controlling PSO

1: / *initialize the swarm.*/

2: **for** *i* = 1 → *PN* **do**

3:  create particle *p*_*i*_ with dimension *D*, velocity *v*_*i*_ and position *x*_*i*_ from 1 to *PN*.

4:  set *x*_*lbest*_(*i*) = *x*_*i*_

5:  calculate *fitness*(*x*_*i*_).

6: **end for**

7: set *x*_*gbest*_ = best(*x*_*lbest*_(*i*))

8: calculate inertia coefficient *w*_*pso*_, cognitive coefficient *c*_1_ and social coefficient *c*_2_.

9: set maximum generation number *MaxT* and chaotic variables *Cr*^0^ and *Cx*^0^.

10: / *update velocity and position with an evolutionary PID style strategy.*/

11: **for** *t* = 1 → *MaxT* **do**

12:  calculate PID controller parameters: *k*_*p*_, *k*_*i*_ *and* *k*_*d*_.

13:  **for** *i* = 1 → *PN* **do**

14:   / *improve local best position at a given generation.*/

15:   calculate velocity *v*_*i*_ and position *x*_*i*_, according to Eqs (2) and (3).

16:   **if** *fitness*(*x*_*i*_)<*fitness*(*x*_*lbest*_(*i*)) **then**

17:    set *x*_*lbest*_(*i*) = *x*_*i*_

18:   **else**

19:    set *repeat*_*num*(*i*) = *repeat*_*num*(*i*) + 1

20:   **end if**

21:   **if** *fitness*(*x*_*lbest*_(*i*)) < *fitness*(*x*_*gbest*_) **then**

22:    set *x*_*gbest*_ = *x*_*lbest*_(*i*)

23:    set *fitness*(*x*_*gbest*_) = *fitness*(*x*_*lbest*_(*i*)

24:   **end if**

25:  **end if**

26:  /*chaotic local search for global best position.*/

27:  If the fitness of the global best position is the same value seven times, then implement the chaotic local search for the global best position, and update the global best position.

28:  /*operation termination.*/

29:  **if** goal threshold or maximum generation number *MaxT* reaches **then**

30:   break

31:  **end if**

32: **end for**

33: output results.

### 2.2 Convergent analysis of chaotic PID controlling PSO

In this part, the convergence of chaotic PID controlling PSO is analytically studied.

**Theorem 1**. *In chaotic PID controlling PSO where its recurrence equations are* Eqs (2) and (3), *when parameters relation* $0<\left(\phi_{1}\left(k_{p}+k_{i}+k_{d}\right)+\phi_{2}\left(k_{p}+k_{i}+k_{d}\right)\right)\leq \left(1+\omega_{pso}-2\sqrt{\left(\omega_{pso}-\phi_{1}\cdot k_{d}-\phi_{2}\cdot k_{d}\right)}\right)$ *or* $\left(1+\omega_{pso}+2\sqrt{\left(\omega_{pso}-\phi_{1}\cdot k_{d}-\phi_{2}\cdot k_{d}\right)}\right)\leq \left(\phi_{1}\left(k_{p}+k_{i}+k_{d}\right)+\phi_{2}\left(k_{p}+k_{i}+k_{d}\right)\right)<\left(2\omega_{pso}+2\right)$ *is satisfied, it is convergent*.

*Proof*. From Eqs (2) and (3), we yield Eq (11).

$$\begin{array}{c} \begin{array}{cc} & x\left(t+1\right)-\left(1+\omega_{pso}-\phi_{1}\cdot \left(k_{p}+k_{i}+k_{d}\right)-\phi_{2}\cdot \left(k_{p}+k_{i}+k_{d}\right)\right)\cdot x\left(t\right)\\ & +\left(\omega_{pso}-\phi_{1}\cdot k_{d}-\phi_{2}\cdot k_{d}\right)\cdot x\left(t-1\right)\\ & =\phi_{1}\cdot \left(k_{p}+k_{i}+k_{d}\right)\cdot x_{lbest}+\phi_{2}\cdot \left(k_{p}+k_{i}+k_{d}\right)\cdot x_{gbest} \end{array} \end{array}$$ (11)

This recurrence equation is approximately constant coefficient nonhomogeneous linear, and the secular equation of the corresponding homogeneous recurrence equation is as follows:

$$\begin{array}{c} f\left(x\right)=x^{2}-\left(1+\omega_{pso}-\phi_{1}\cdot \left(k_{p}+k_{i}+k_{d}\right)-\phi_{2}\cdot \left(k_{p}+k_{i}+k_{d}\right)\right)\cdot x+\left(\omega_{pso}-\phi_{1}\cdot k_{d}-\phi_{2}\cdot k_{d}\right)=0. \end{array}$$ (12)

Supposing *K* = (*k*_*p*_ + *k*_*i*_ + *k*_*d*_), the latent roots of the above secular equation are as follows:

$$\begin{array}{c} \begin{array}{c} x_{1}=\frac{1+\omega_{pso}-\phi_{1}\cdot K-\phi_{2}\cdot K+\sqrt{{\left(1+\omega_{pso}-\phi_{1}\cdot K-\phi_{2}\cdot K\right)}^{2}-4\cdot \left(\omega_{pso}-\phi_{1}\cdot k_{d}-\phi_{2}\cdot k_{d}\right)}}{2},\\ x_{2}=\frac{1+\omega_{pso}-\phi_{1}\cdot K-\phi_{2}\cdot K-\sqrt{{\left(1+\omega_{pso}-\phi_{1}\cdot K-\phi_{2}\cdot K\right)}^{2}-4\cdot \left(\omega_{pso}-\phi_{1}\cdot k_{d}-\phi_{2}\cdot k_{d}\right)}}{2}. \end{array} \end{array}$$ (13)

According to the relations of the recurrence Eq (11) and its special solution, we can solve the special solution below.

$$\begin{array}{c} x^{*}=\frac{\phi_{1}\cdot \left(k_{p}+k_{i}+k_{d}\right)\cdot x_{lbest}+\phi_{2}\cdot \left(k_{p}+k_{i}+k_{d}\right)\cdot x_{gbest}}{\left(k_{p}+k_{i}\right)\left(\phi_{1}+\phi_{2}\right)} \end{array}$$ (14)

According to the relations of the recurrence Eq (11), its general solution, special solution, and latent roots, we can obtain the general solution of the recurrence Eq (11) as follows:

$$\begin{array}{c} x\left(t\right)=x^{*}+C_{1}\cdot x_{1}^{t}+C_{2}\cdot x_{2}^{t}. \end{array}$$ (15)

Evidently, if there exist *f*(1) > 0, *f*(−1) > 0, and (1 + *ω*_*pso*_ − *ϕ*_1_ ⋅ *K* − *ϕ*_2_ ⋅ *K*)^2^ − 4 ⋅ (*ω*_*pso*_ − *ϕ*_1_ ⋅ *k*_*d*_ − *ϕ*_2_ ⋅ *k*_*d*_) ≥ 0, then there are −1 < *x*_1_ < 1 and −1 < *x*_2_ < 1. Namely, if $0<\left(\phi_{1}\left(k_{p}+k_{i}+k_{d}\right)+\phi_{2}\left(k_{p}+k_{i}+k_{d}\right)\right)\leq \left(1+\omega_{pso}-2\sqrt{\left(\omega_{pso}-\phi_{1}\cdot k_{d}-\phi_{2}\cdot k_{d}\right)}\right)$ or $\left(1+\omega_{pso}+2\sqrt{\left(\omega_{pso}-\phi_{1}\cdot k_{d}-\phi_{2}\cdot k_{d}\right)}\right)\leq \left(\phi_{1}\left(k_{p}+k_{i}+k_{d}\right)+\phi_{2}\left(k_{p}+k_{i}+k_{d}\right)\right)<\left(2\omega_{pso}+2\right)$ is satisfied, then we can obtain −1 < *x*_1_ < 1 and −1 < *x*_2_ < 1. Such indicates that if −1 < *x*_1_ < 1 and −1 < *x*_2_ < 1 hold, then we can finalize the limit of x(t), namely

$$\begin{array}{c} \begin{array}{cc} \lim_{t\to +\infty }x\left(t\right) & =\lim_{t\to +\infty }\left(x^{*}+C_{1}\cdot x_{1}^{t}+C_{2}\cdot x_{2}^{t}\right)\\ & =\lim_{t\to +\infty }\left(x^{*}\right)\\ & =\frac{\phi_{1}\cdot x_{lbest}+\phi_{2}\cdot x_{gbest}}{\left(\phi_{1}+\phi_{2}\right)}. \end{array} \end{array}$$ (16)

Therefore, the above **Theorem 1**. is existent.

## 3 Experimental study

In this part, we conduct a detailed experimental study to evaluate the performance of chaotic PID controlling PSO. The experiments include the description of the experimental setup, convergence, robustness, computational cost of chaotic PID controlling PSO as well as experimental discussion.

### 3.1 Description of the experimental setup

#### 3.1.1 Selected chaotic PSO algorithms and parameter setting

In order to illustrate, compare and analyze the effectiveness and performance of chaotic PID controlling PSO, we select four state-of-the-art chaotic PSO variants including the proposed chaotic PID controlling PSO to conduct the experiments on the ten analytic test problems with 5, 15 and 100 dimensions. These chaotic PSO variants are listed below and their settings of important parameters are summarized in Table 1.

- Chaotic PSO with the logistic map (CPSO-1) [6];
- Chaotic PSO with the tent map (CPSO-2) [7];
- Chaotic catfish PSO with the logistic map (CPSO-3) [11];
- Chaotic PID controlling PSO (CPIDSO).

**Table 1:** Parameters settings for involved chaotic PSO algorithms.

Name	Inertia Weight	Acceleration Coefficients and Others
CP_ID_SO	*w*_*pso*_ is decided by Eq (7) where *w*_*pso*_*min*__ = 0.4, *w*_*pso*_*max*__ = 0.9.	$c_{1}\left(t\right)=2.0-\frac{2.0\cdot t}{MaxT}\;c_{2}\left(t\right)=\frac{2.0\cdot t}{MaxT}\;k_{p}=e^{\left(w_{pso}-1\right)\cdot \frac{t}{MaxT}}\;k_{i}=\frac{e^{\left(w_{pso}-1\right)\cdot \frac{t}{MaxT}}}{1+e^{\left(w_{pso}-1\right)\cdot \frac{t}{MaxT}}}\;k_{d}={\left[e^{\left(w_{pso}-1\right)\cdot \frac{t}{MaxT}}\right]}^{2}$
CPSO-1	*w*_*pso*_ is decided by Eq (7) where *w*_*pso*_*min*__ = 0.05, *w*_*pso*_*max*__ = 1.05.	*c*_1_(*t*) = *c*_2_(*t*) = 2.05
CPSO-2	*w*_*pso*_ is decided by Eq (7) where *w*_*pso*_*min*__ = 0.05, *w*_*pso*_*max*__ = 1.05.	*c*_1_(*t*) = *c*_2_(*t*) = 2.05
CPSO-3	$w_{pso}\left(t\right)=0.9-\frac{0.5\cdot t}{MaxT}$	*c*_1_(*t*) = *c*_2_(*t*) = 2.05

#### 3.1.2 Benchmark functions

**Fig 1:**
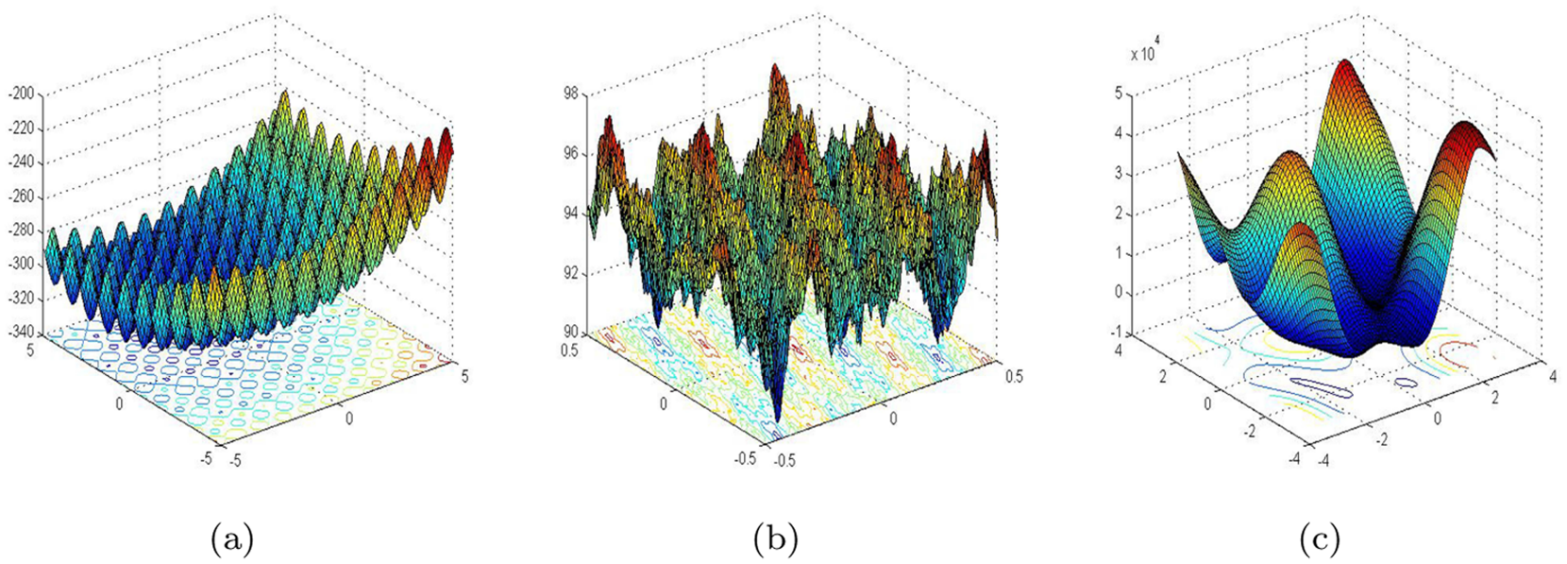
The 3-D maps for 2-D test functions *f*_3_, *f*_5_ *and* *f*_6_. (a) Shifted Rastrigin’s Function. (b) Shifted Rotated Weierstrass Function. (c) Schwefel’s Problem 2.13.

Ten representative benchmark functions are used to test the selected chaotic PSO algorithms [29]. They are shown below in Table 2. Since chaos attempts to help evolutionary algorithms avoid getting stuck in local optima, these benchmark functions is mainly considered to be multimodal problems. It is apparent that most of these test functions are the hybrid composites of the typical multimodal functions like Ackley, Rosenbrock, Griewank, Rastrigin, Schwefel and Weierstrass functions so that their properties become more complicated and much closer to the environments in the real world. Thus such are beneficial to the reasonable verification of performance evaluation of chaotic PID controlling PSO. Three dimensional maps for two dimensional test functions *f*_3_, *f*_5_ *and* *f*_6_ in Table 2 are shown in Fig 1.

**Table 2:** Selected analytic benchmark functions for performance testing of diverse chaotic PSO algorithms.

Name	Test Function	Dimensionality	Search Range	Global Minimum	Modality
Shifted Rosenbrock’s Function	$f_{1}\left(x\right)=\sum_{i=1}^{D-1}\left(100{\left(z_{i}-z_{i+1}\right)}^{2}+{\left(z_{i}-1\right)}^{2}\right)+390,z=x-o+1$, *o* is the shift global optimum.	[5, 100]	[−100, 100]^*D*^	390	Multimodal
Shifted Rotated Ackley’s Function with Global Optimum on Bounds	$f_{2}\left(x\right)=-20\text{exp}\left(-0.2\sqrt{1/D\sum_{i=1}^{D}x_{i}^{2}}\right)-\text{exp}\left(1/D\sum_{i=1}^{D}\text{cos}\left(2\pi x_{i}\right)\right)+20+e-140,z=\left(x-o\right)\times M$, *o* is the shift global optimum, *M* is the linear transformation matrix with condition number 100.	[5, 100]	[−32, 32]^*D*^	-140	Multimodal
Shifted Rastrigin’s Function	$f_{3}\left(x\right)=\sum_{i=1}^{D}\left(z_{i}^{2}+10\text{cos}\left(2\pi z_{i}\right)+10\right)-330,z=x-o$, *o* is the shift global optimum.	[5, 100]	[−5, 5]^*D*^	-330	Multimodal
Shifted Rotated Rastrigin’s Function	$f_{4}\left(x\right)=\sum_{i=1}^{D}\left(z_{i}^{2}+10\text{cos}\left(2\pi z_{i}\right)+10\right)-330,z=\left(x-o\right)\times M$, *o* is the shift global optimum, *M* is the linear transformation matrix with condition number 2.	[5, 100]	[−5, 5]^*D*^	-330	Multimodal
Shifted Rotated Weierstrass Function	$f_{5}\left(x\right)=\sum_{i=1}^{D}\left\{\sum_{k=0}^{kmax}\left[a^{k}\text{cos}\left(2\pi b^{k}\left(z_{i}+0.5\right)\right)\right]\right\}-D\sum_{k=0}^{kmax}\left[a^{k}\text{cos}\left(2\pi b^{k}\cdot 0.5\right)\right]+90,a=0.5,b=3,kmax=20,z=\left(x-o\right)\times M$, *o* is the shift global optimum, *M* is the linear transformation matrix with condition number 5.	[5, 100]	[−0.5, 0.5]^*D*^	90	Multimodal
Schwefel’s Problem 2.13	$f_{6}\left(x\right)=\sum_{i=1}^{D}{\left(A_{i}-B_{i}\left(x\right)\right)}^{2}-460,A_{i}=\sum_{j=1}^{D}\left(a_{ij}\text{sin}\alpha_{j}+b_{ij}\text{cos}\alpha_{j}\right),B_{i}\left(x\right)=\sum_{j=1}^{D}\left(a_{ij}\text{sin}x_{j}+b_{ij}\text{cos}x_{j}\right)$, *A*, *B* are two *D* × *D* matrices, *a*_*ij*_, *b*_*ij*_ are the integer random numbers in the range [−100, 100], *α* = [*α*_1_, *α*_2_, ⋯, *α*_*D*_], *α*_*j*_ is the random number in the range [−*π*, *π*].	[5, 100]	[−*π*, *π*]^*D*^	-460	Multimodal
Expanded Extended Griewank’s plus Rosenbrock’s Function (G(R(x)))	Griewank’s Function $:G\left(x\right)=1/4000\sum_{i=1}^{D}x_{i}^{2}-\prod_{i=1}^{D}\text{cos}\left(x_{i}/\sqrt{i}\right)+1$, Rosenbrock’s Function $:R\left(x\right)=\sum_{i=1}^{D-1}\left(100{\left(x_{i}^{2}-x_{i+1}\right)}^{2}+{\left(x_{i}-1\right)}^{2}\right)$, *f*_7_(*x*) = *G*(*R*(*z*_1_, *z*_2_))+*G*(*R*(*z*_2_, *z*_3_)) + ⋯+*G*(*R*(*z*_*D*−1_, *z*_*D*_))+*G*(*R*(*z*_*D*_, *z*_1_))−130, *z* = *x* − *o*+1, *o* is the shift global optimum.	[5, 100]	[−5, 5]^*D*^	-130	Multimodal
Shifted Rotated Expanded Scaffer’s SF(x) Function	$SF\left(x\right)=0.5+\frac{{\text{sin}}^{2}\left(\sqrt{\left(x^{2}+y^{2}\right)}\right)-0.5}{1+0.001\left(x^{2}+y^{2}\right){)}^{2}},f_{8}\left(x\right)=SF\left(z_{1},z_{2}\right)+SF\left(z_{2},z_{3}\right)+\ldots +SF\left(z_{D-1},z_{D}\right)+SF\left(z_{D},z_{1}\right)-300,z=\left(x-o\right)\times M$, *o* is the shift global optimum, *M* is the linear transformation matrix with condition number 3.	[5, 100]	[−100, 100]^*D*^	-300	Multimodal
Hybrid Composition Function 1	*f*_1−2_: Rastrigin’s Function, *f*_3−4_: Weierstrass Function, *f*_5−6_: Griewank’s Function, *f*_7−8_: Ackley’s Function, *f*_9−10_: Sphere Function, $f_{9}\left(x\right)=\sum_{i=1}^{10}f_{i}\left(z\right)+120,z=\left(\left(x-o_{i}\right)/\lambda_{i}\right)\times M_{i})$, *σ*_*i*_ = 1 (*i* = 1, 2, ⋯, *D*), *λ* = [1; 1; 10; 10; 5/60; 5/60; 5/32; 5/32; 5/100; 5/100], *M*_*i*_ is the identity matrix.	[5, 100]	[−5, 5]^*D*^	120	Multimodal
Rotated Hybrid Composition Function 1	All other settings in *f*_10_ are the same as *f*_9_ except *M*_*i*_ is the different linear transformation matrix with condition number 2.	[5, 100]	[−5, 5]^*D*^	120	Multimodal

### 3.2 Convergence of chaotic PID controlling PSO

In order to validate the convergent performance of chaotic PID controlling PSO, chaotic PID controlling PSO together with other three chaotic PSO algorithms are conducted on the benchmark test functions in Table 2. When the 5-dimensional (5-D) problems are solved, the population size is set at 15 and the maximum fitness evaluations (FEs) is set at 15000. When the 15-dimensional (15-D) problems are solved, the population size is set at 25 and the maximum FEs is set at 50000. When the 100-dimensional (100-D) problems are solved, the population size is set at 100 and the maximum FEs is set at 200000. All experiments are run 20 times. The mean values, standard deviation of the results, and the best values are presented. And in order to determine whether the results obtained by chaotic PID controlling PSO are statistically different from the results generated by other chaotic PSO variants, the nonparametric Wilcoxon rank sum tests are conducted between the chaotic PID controlling PSO’s result and the result achieved by the other chaotic PSO algorithms for each test function.

**Fig 2:**
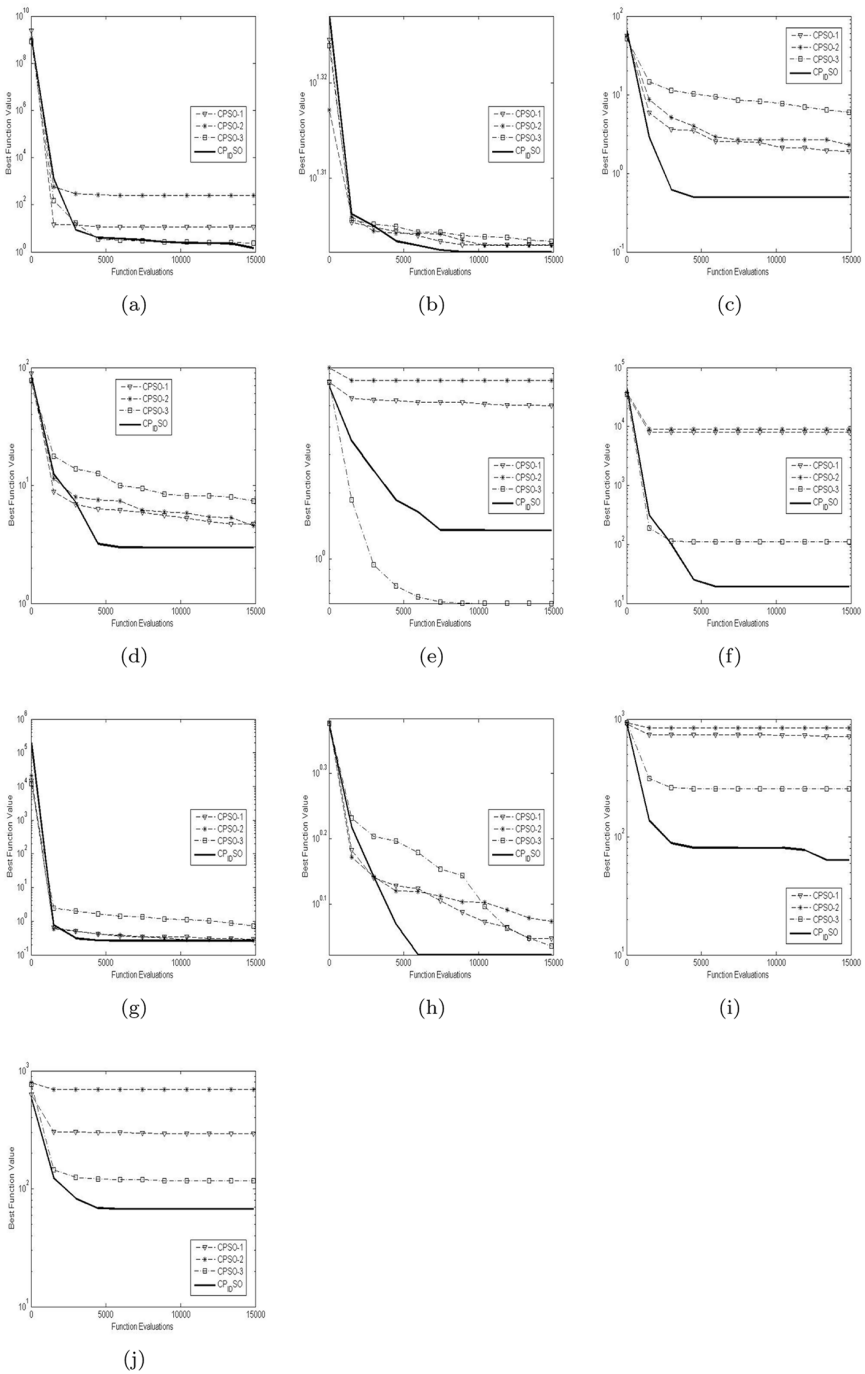
The median convergence characteristics of diverse chaotic PSO algorithms for the 5-D test functions. (a) Shifted Rosenbrock’s function. (b) Shifted rotated Ackley’s function with global optimum on bounds. (c) Shifted Rastrigin’s function. (d) Shifted rotated Rastrigin’s function. (e) Shifted rotated Weierstrass function. (f) Schwefel’s problem 2.13. (g) Expanded extended Griewank’s plus Rosenbrock’s function (G(R(x))). (h) Shifted rotated expanded Scaffer’s SF(x) function. (i) Hybrid composition function 1. (j) Rotated hybrid composition function 1.

**Fig 3:**
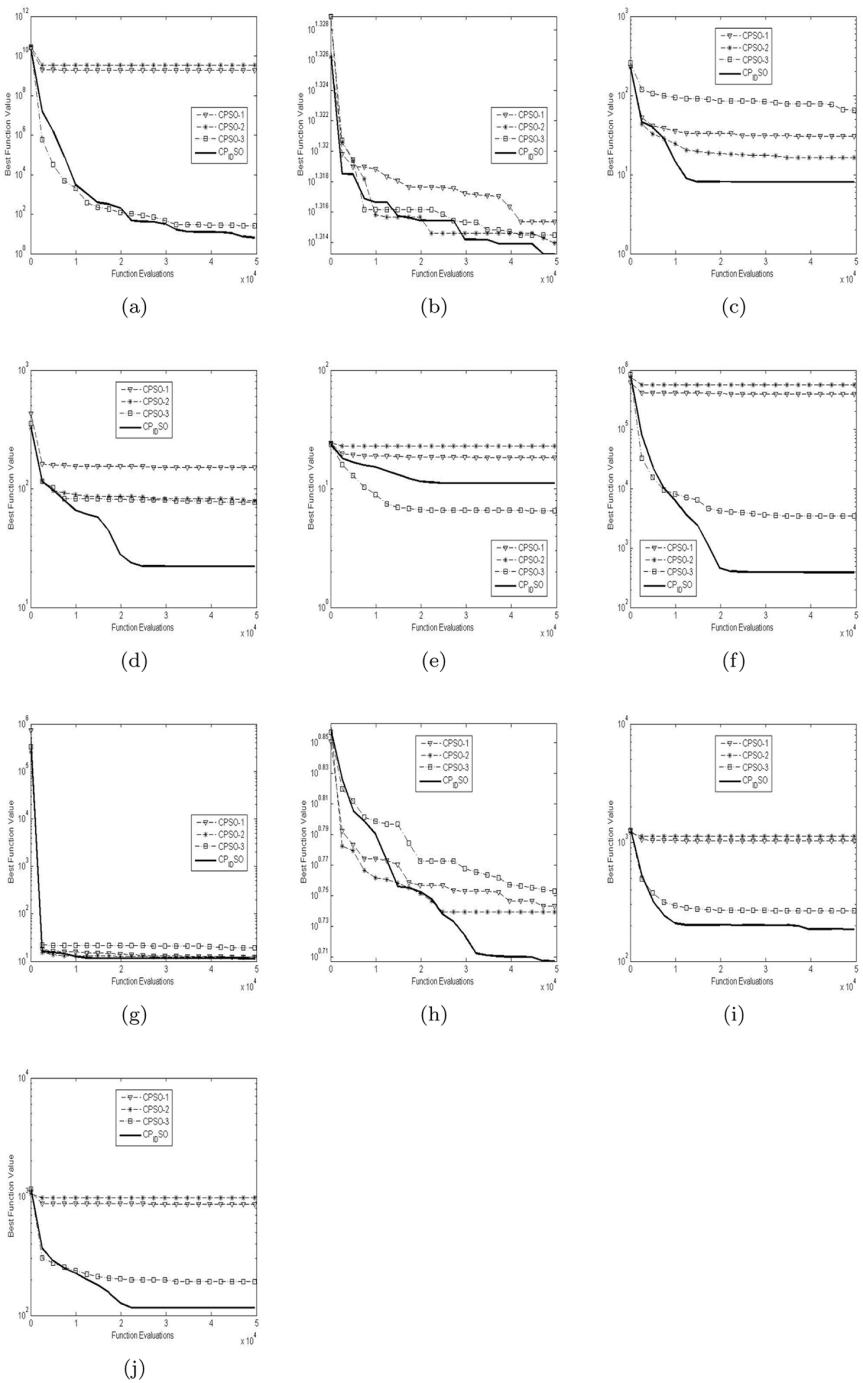
The median convergence characteristics of diverse CPSO algorithms for the 15-D test functions. (a) Shifted Rosenbrock’s function. (b) Shifted rotated Ackley’s function with global optimum on bounds. (c) Shifted Rastrigin’s function. (d) Shifted rotated Rastrigin’s function. (e) Shifted rotated Weierstrass function. (f) Schwefel’s problem 2.13. (g) Expanded extended Griewank’s plus Rosenbrock’s function (G(R(x))). (h) Shifted rotated expanded Scaffer’s SF(x) function. (i) Hybrid composition function 1. (j) Rotated hybrid composition function 1.

**Fig 4:**
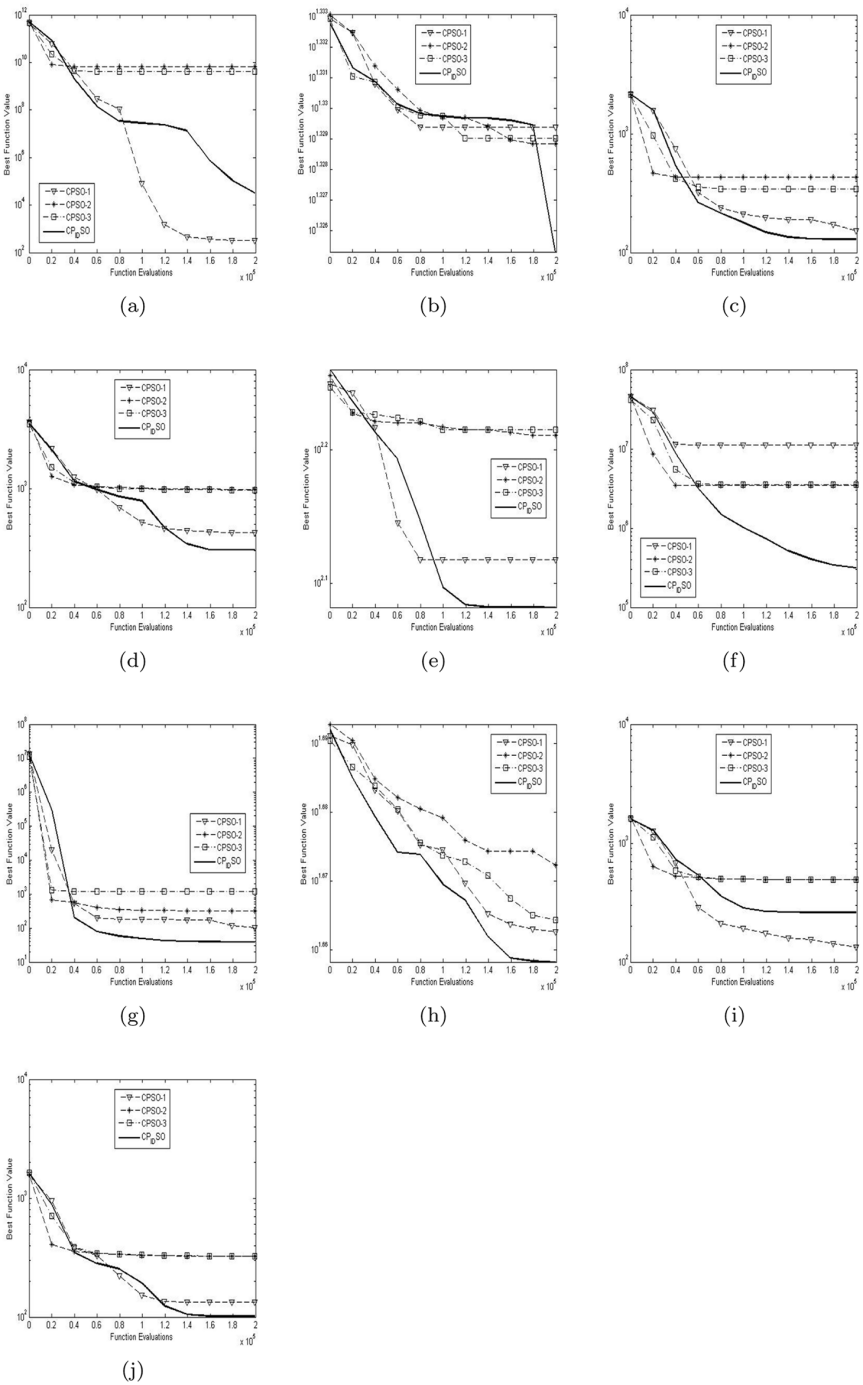
The median convergence characteristics of diverse CPSO algorithms for the 100-D test functions. (a) Shifted Rosenbrock’s function. (b) Shifted rotated Ackley’s function with global optimum on bounds. (c) Shifted Rastrigin’s function. (d) Shifted rotated Rastrigin’s function. (e) Shifted rotated Weierstrass function. (f) Schwefel’s problem 2.13. (g) Expanded extended Griewank’s plus Rosenbrock’s function (G(R(x))). (h) Shifted rotated expanded Scaffer’s SF(x) function. (i) Hybrid composition function 1. (j) Rotated hybrid composition function 1.

Table 3 presents the global minimum means and variances of the 20 runs of the four chaotic PSO algorithms on the ten test functions with their dimensions 5 in Table 2. Table 4 presents the global minimum means and variances of the 20 runs of the four chaotic PSO algorithms on the ten test functions with their dimensions 15 in Table 2. Table 5 presents the global minimum means and variances of the 20 runs of the four chaotic PSO algorithms on the ten test functions with their dimensions 100 in Table 2. The best results among the four chaotic PSO algorithms are shown in bold in Tables 3–5. Fig 2 presents the convergence characteristics in terms of the best fitness value of the median run of diverse chaotic PSO algorithms for each test function with its dimension 5. Fig 3 presents the convergence characteristics in terms of the best fitness value of the median run of diverse chaotic PSO algorithms for each test function with its dimension 15. Fig 4 presents the convergence characteristics in terms of the best fitness value of the median run of diverse chaotic PSO algorithms for each test function with its dimension 100. The results of the proposed chaotic PID controlling PSO are depicted by bold solid lines in Figs 2, 3 and 4. Note that the function fitness here is defined as the absolute value of given global minimum in Table 2 minus computed global minimum. And the approximate results of Y axises in Figs 2, 3 and 4 are logarithmic.

**Table 3:** Computed global minimum results of diverse chaotic PSO algorithms for the 5-D multimodal problems.

Function		CPSO-1	CPSO-2	CPSO-3	CP_ID_SO	h_t-tests
*f*_1_	Mean	401.1576	636.8849	392.3767	**391.4706**	1
	Std. Dev	28.6014	428.4596	3.3729	**2.0146**	
	Best	390.4399	390.0826	390.0023	**390.0014**	
*f*_2_	Mean	-119.9107	-119.9121	-119.8939	**-119.9444**	1
	Std. Dev	0.0457	0.0719	0.0593	**0.0404**	
	Best	-119.9749	-119.9556	-119.9476	**-119.9997**	
*f*_3_	Mean	-328.0900	-327.6960	-324.0352	**-329.5025**	1
	Std. Dev	1.0834	1.7562	2.48211	**0.8456**	
	Best	-330.0000	-330.0000	-326.8923	**-330.0000**	
*f*_4_	Mean	-325.2766	-325.6341	-322.5742	**-327.0151**	1
	Std. Dev	1.0430	1.6878	2.3625	**1.4071**	
	Best	-326.4124	-328.6842	-326.7826	**-329.0050**	
*f*_5_	Mean	95.0370	95.9475	**90.2708**	91.0640	1
	Std. Dev	1.0389	0.9939	**0.5996**	0.8440	
	Best	92.8446	**94.5356**	90.0000	90.1649	
*f*_6_	Mean	7.5301e+003	8.5595e+003	-349.4718	**-440.8349**	1
	Std. Dev	8.2880e+003	8.2085e+003	200.0335	**60.6052**	
	Best	-128.2554	1.0322e+003	-460.0000	**-460.0000**	
*f*_7_	Mean	-129.7549	-129.7339	-129.3167	**-129.7623**	1
	Std. Dev	0.0978	0.1580	0.1876	**0.1815**	
	Best	-129.8599	-129.9901	-129.6263	**-129.8735**	
*f*_8_	Mean	-298.8860	-298.8154	-298.9158	**-298.9485**	1
	Std. Dev	0.3045	0.3322	0.3541	**0.3539**	
	Best	-299.4441	-299.4241	-299.5137	**-299.8145**	
*f*_9_	Mean	831.5733	958.5152	375.6961	**183.7124**	1
	Std. Dev	115.3580	173.4461	293.9829	**64.7185**	
	Best	667.8437	669.4214	120.0000	**120.0000**	
*f*_10_	Mean	541.9082	745.8038	252.7339	**187.3590**	1
	Std. Dev	182.3725	179.8922	31.0163	**38.2756**	
	Best	318.2975	523.5829	220.0000	**120.0000**	

**Table 4:** Computed global minimum results of diverse chaotic PSO algorithms for the 15-D multimodal problems.

Function		CPSO-1	CPSO-2	CPSO-3	CP_ID_SO	h_t-tests
*f*_1_	Mean	1.9032e+009	3.4992e+009	416.1978	**396.3656**	1
	Std. Dev	2.7771e+009	6.2945e+009	38.8779	**10.5258**	
	Best	4.1432e+006	3.4563e+006	395.0309	**390.0036**	
*f*_2_	Mean	-119.3287	-119.3950	-119.3705	**-119.4288**	1
	Std. Dev	0.1530	0.0997	0.0772	**0.0879**	
	Best	-119.5707	-119.5000	-119.4989	**-119.5528**	
*f*_3_	Mean	-299.6025	-313.7158	-264.7374	**-321.8413**	1
	Std. Dev	15.7024	8.3512	8.9379	**3.9423**	
	Best	-315.0451	-325.0252	-277.4704	**-326.0202**	
*f*_4_	Mean	-178.2664	-250.5255	-252.6327	**-307.6782**	1
	Std. Dev	118.3594	31.2863	7.7686	**6.5647**	
	Best	-279.2571	-274.5268	-263.4672	**-315.0756**	
*f*_5_	Mean	108.2593	112.7917	**96.5544**	101.1674	1
	Std. Dev	2.0914	1.8639	**1.4592**	1.2259	
	Best	103.8539	109.6547	**94.4507**	99.0730	
*f*_6_	Mean	3.4319e+005	6.4646e+005	1.3867e+003	**-67.9555**	1
	Std. Dev	1.3521e+005	8.8279e+004	2.3977e+003	**579.7256**	
	Best	1.6848e+005	5.3627e+005	-377.6398	**-459.8871**	
*f*_7_	Mean	-127.6425	-127.7221	-121.2105	**-128.6958**	1
	Std. Dev	1.0311	0.5360	1.5602	**0.2173**	
	Best	-128.6055	-128.3805	-122.9334	**-128.9892**	
*f*_8_	Mean	-294.4624	-294.5116	-294.3698	**-294.9037**	1
	Std. Dev	0.2168	0.3421	0.1356	**0.5616**	
	Best	-294.7204	-294.9652	-294.5878	**-295.8638**	
*f*_9_	Mean	1.1498e+003	1.2466e+003	387.4979	**306.5345**	1
	Std. Dev	145.3173	107.4211	146.8314	**164.0320**	
	Best	956.7701	1.0109e+003	194.6305	**166.1034**	
*f*_10_	Mean	979.8030	1.1016e+003	311.6831	**236.0910**	1
	Std. Dev	208.5678	94.0947	109.2600	**33.8973**	
	Best	657.0202	965.9093	256.1366	**189.9781**	

**Table 5:** Computed global minimum results of diverse chaotic PSO algorithms for the 100-D multimodal problems.

Function		CPSO-1	CPSO-2	CPSO-3	CP_ID_SO	h_t-tests
*f*_1_	Mean	**716.9861**	6.4991e+009	4.2684e+009	3.3448e+004	0
	Std. Dev	**73.3234**	2.4254e+009	3.8121e+008	5.5519e+004	
	Best	**651.8337**	3.8443e+009	4.0386e+009	1.1057e+003	
*f*_2_	Mean	-118.6506	-118.6768	-118.6678	**-118.9304**	1
	Std. Dev	0.0134	0.0091	0.0207	**0.0450**	
	Best	-118.6622	-118.6870	-118.6915	**-118.9761**	
*f*_3_	Mean	-178.6908	103.8945	11.4895	**-200.1354**	1
	Std. Dev	15.5659	27.6276	134.5585	**19.5557**	
	Best	-195.5331	72.0069	-136.9559	**-214.0199**	
*f*_4_	Mean	93.0259	645.0898	641.0177	**-26.1216**	1
	Std. Dev	168.1029	53.8996	37.1054	**54.7147**	
	Best	-84.2456	600.7580	598.4596	**-69.3212**	
*f*_5_	Mean	221.1097	252.5685	254.1262	**210.8574**	1
	Std. Dev	7.8295	1.8075	1.6344	**16.8435**	
	Best	212.2674	250.5724	252.4688	**191.5652**	
*f*_6_	Mean	1.1092e+007	3.4811e+006	3.5245e+006	**3.1488e+005**	1
	Std. Dev	1.1215e+007	5.5381e+005	3.0153e+005	**8.3980e+004**	
	Best	2.6803e+006	2.8916e+006	3.1929e+006	**2.4195e+005**	
*f*_7_	Mean	-29.1531	194.47921	1.0417e+003	**-91.0219**	1
	Std. Dev	26.6782	299.0761	835.9888	**8.3322**	
	Best	-48.0170	-84.8112	385.9904	**-100.6265**	
*f*_8_	Mean	-254.0078	-252.9893	-253.8350	**-254.4749**	1
	Std. Dev	1.1289	0.5403	0.1247	**0.5932**	
	Best	-255.1097	-253.5232	-253.9397	**-255.1205**	
*f*_9_	Mean	**253.5445**	612.4794	611.2137	381.7007	0
	Std. Dev	**22.1042**	76.1553	92.1949	13.8517	
	Best	**228.0567**	565.8306	507.0778	366.1607	
*f*_10_	Mean	253.0357	445.4767	444.8349	**221.7206**	1
	Std. Dev	19.3403	4.7021	3.0445	**10.7733**	
	Best	234.9268	440.0476	441.8650	**212.2127**	

From the results in Table 3, we clearly observe that for the multimodal problems in Table 2, chaotic PID controlling PSO achieves best results on most of the test functions *f*_1_−*f*_4_ *and* *f*_6_−*f*_7_ while it does not exhibit the best performance on the test function *f*_5_. In addition, chaotic PID controlling PSO performs better than CPSO-1 and CPSO-2 on the function *f*_5_, but CPSO-3 achieves the best result on the test function *f*_5_. It is worth noting that compared with CPSO-1 and CPSO-2, CPSO-3 yields comparatively better results on the test functions *f*_1_, *f*_6_, *f*_8_, *f*_9_ *and* *f*_10_. Furthermore, CPSO-1 performs better than CPSO-2 and CPSO-3 on the test functions *f*_3_ *and* *f*_7_ whilst CPSO-2 does better than CPSO-1 and CPSO-3 on the test functions *f*_2_ *and* *f*_4_. Comparing the results in Table 3 with the graphs in Fig 2, we find out that CPSO-1 and CPSO-2 perform rather worse on the test functions *f*_6_, *f*_9_ *and* *f*_10_ and CPSO-3 does worst on the test functions *f*_2_, *f*_3_, *f*_4_ *and* *f*_7_. Such chaotic PID controlling PSO’s results demonstrate its better effectiveness and efficiency on solving most multimodal problems.

When the dimensional size increases from 5 to 15, the experiments similar to those conducted on the 5-D problems are repeated on the 15-D problems, and the results and graphs are presented in Table 4 and Fig 3. From the results in Table 4 and the graphs in Fig 3, we observe that though the results of the diverse chaotic PSO algorithms for the 15-D problems in Table 2 are not as comparatively good as those for the 10-D problems, the diverse chaotic PSO algorithms for the 15-D problems have many similarities as those for the 5-D problems. chaotic PID controlling PSO still exhibits best results on most of the test functions *f*_1_−*f*_4_ *and* *f*_6_−*f*_7_ except *f*_5_ while CPSO-3 achieves the best result on the test function *f*_5_. Besides, CPSO-1 and CPSO-2 perform worse on the test functions *f*_1_, *f*_6_, *f*_9_ *and* *f*_10_ and CPSO-3 still achieves the worst results on the test functions *f*_3_ *and* *f*_7_. However, despite these results, CPSO-2 and CPSO-3 become robust to conduct on the complex problems in Table 2 as the dimensional size increases from 5 to 15. From the graphs in Fig 3, it is obvious that chaotic PID controlling PSO shows much better results than do other CPSO algorithms on most complex multimodal problems since the time varying PID controller, chaotic random parameters and chaotic local search for the global best position have effectively improved the evolutionary dynamics of particles at the same time.

From the graphs in Fig 4 and the results in Table 5, it can be observed that when the high 100-D problems are solved, diverse chaotic PSO algorithms sharply degenerate. Although chaotic PID controlling PSO still achieves the best results on most of the test functions, its search capability obviously get weaker than before so that it suffers from local optimal and degeneracy problem, especially on the test function *f*_6_. There are several important causes to merit attention. Besides the usual search space expansion, the lacks of more effective social learning, hierarchical inertia weight, chaotic local search strategies are non-negligible ones. Such causes directly result in the deterioration of the diversity of swarm.

### 3.3 Robustness of chaotic PID controlling PSO

Table 6 presents the fixed accuracy level of the selected analytic test functions in Table 2 for performance testing of diverse chaotic PSO algorithms. A successful run denotes the run during which the algorithm achieves the fixed accuracy level within the Maximum FEs for a particular dimension. Based on successful runs, success rate (*Suc*. *Rate*) and success performance (*Suc*. *Perf*.) are defined below [30].

$$\begin{array}{c} Suc.\;Rate=\frac{Successful\;runs}{Total\;runs} \end{array}$$ (17)

$$\begin{array}{c} Suc.\;Perf.=mean\left(FEs\;for\;successful\;runs\right)\cdot \frac{Total\;runs}{Successful\;runs} \end{array}$$ (18)

**Table 6:** Fixed accuracy level of the selected analytic test functions in Table 2.

Function	Accuracy	Function	Accuracy
*f*_1_	390+390 × 0.5%	*f*_6_	−460+460 × 3.5%
*f*_2_	−140+140 × 14.6%	*f*_7_	−130+130 × 0.5%
*f*_3_	−330+330 × 1.5%	*f*_8_	−300+300 × 0.5%
*f*_4_	−330+330 × 1.5%	*f*_9_	120+120 × 2.5%
*f*_5_	90+90 × 3.5%	*f*_10_	120+120 × 2.5%

Table 7 presents the success rates and success performances of diverse chaotic PSO algorithms for the 5-D test functions in Table 2. The best results among the chaotic PSO algorithms are shown in bold in Table 6. From the results in Table 7, it can be seen that chaotic PID controlling PSO achieves the best success rates and success performances when solving the test functions *f*_1_ − *f*_4_ *and* *f*_6_ while CPSO-3 does best on the test function *f*_5_ and CPSO-1 does best on the test functions *f*_7_ *and* *f*_8_. All the Chaotic PSO algorithms do not show good results on the test functions *f*_9_ *and* *f*_10_. However, from the results in Tables 3, 6 and 7, one may conclude that compared with other chaotic PSO algorithms, chaotic PID controlling PSO search the global optima with high successful probability and is comparably more effective and reliable for solving most complex problems in Table 2.

**Table 7:** Success rates and success performances of diverse chaotic PSO algorithms for the 5-D test functions in Table 2.

Function		CPSO-1	CPSO-2	CPSO-3	CP_ID_SO
*f*_ 1_	Suc. Rate	10%	10%	40%	**90%**
	Suc. Perf.	9.5644e+002	8.7950e+003	1.4216e+004	**1.2654e+004**
*f*_ 2_	Suc. Rate	N/A	N/A	N/A	**40%**
	Suc. Perf.	N/A	N/A	N/A	**1.4352e+004**
*f*_ 3_	Suc. Rate	10%	10%	40%	**100%**
	Suc. Perf.	1.1316e+004	1.3197e+004	1.4456e+004	**2.3650e+003**
*f*_ 4_	Suc. Rate	70%	50%	20%	**90%**
	Suc. Perf.	1.3939e+004	1.0522e+004	1.4573e+004	**3.3850e+003**
*f*_ 5_	Suc. Rate	10%	N/A	**100%**	100%
	Suc. Perf.	1.2675e+004	N/A	**1.5140e+003**	3.4832e+003
*f*_ 6_	Suc. Rate	N/A	N/A	50%	**70%**
	Suc. Perf.	N/A	N/A	3.3590e+003	**4.8230+003**
*f*_ 7_	Suc. Rate	**100%**	100%	50%	100%
	Suc. Perf.	**1.4890e+003**	2.8700e+003	1.1631e+004	2.5876e+003
*f*_ 8_	Suc. Rate	**100%**	70%	80%	90%
	Suc. Perf.	**2.9980e+003**	2.2610e+003	6.0350e+003	2.4195e+003
*f*_ 9_	Suc. Rate	N/A	N/A	N/A	N/A
	Suc. Perf.	N/A	N/A	N/A	N/A
*f*_10_	Suc. Rate	N/A	N/A	N/A	N/A
	Suc. Perf.	N/A	N/A	N/A	N/A

### 3.4 Computational cost of chaotic PID controlling PSO

To investigate the computational cost of chaotic PID controlling PSO, the four chaotic PSO algorithms are required to conduct the experiments on the test functions with 100-D size in Table 2. The population size is set at 100 and the maximum FEs is set at 200000. Besides, all experiments are run 20 times, when using MATLAB 7.80, Windows 7.0, 4 GByte RAM, Intel Core i7-2820QM 2.30 GHz processor. Table 8 gives the average computational cost time (seconds) of diverse chaotic PSO algorithms for the test functions *f*_1_ − *f*_10_ with 100-D size. The best results among the four chaotic PSO algorithms are shown in bold in Table 8.

**Table 8:** The average computational cost time (seconds) of diverse chaotic PSO algorithms for the test functions *f*_1_ − *f*_10_ with 100-D size.

Function	CPSO-1	CPSO-2	CPSO-3	CP_ID_SO
*f*_1_	823.9988	**388.0771**	490.7063	677.3888
*f*_2_	425.6647	**199.8957**	312.4547	542.5901
*f*_3_	733.5355	631.2889	**606.2011**	830.3098
*f*_4_	474.3410	454.0797	**232.5310**	562.6557
*f*_5_	872.5215	658.6773	**342.9804**	1.0224e+003
*f*_6_	**64.6663**	772.7345	258.1963	427.3578
*f*_7_	**908.2889**	995.0521	964.4901	1.1524e+003
*f*_8_	560.8061	**318.7199**	544.9402	662.9074
*f*_9_	3.2899e+003	**1.4951e+003**	1.8800e+003	3.0372e+003
*f*_10_	2.8123e+003	1.8717e+003	**1.2642e+003**	5.7762e+003

From the computational cost results in Table 8, CPSO-3 consumes least on the test functions *f*_3_, *f*_4_, *f*_5_, *f*_10_ whilst CPSO-2 does least on the test functions *f*_1_, *f*_2_, *f*_8_, *f*_9_, and CPSO-1 does least on the test functions *f*_6_, *f*_7_. The computational cost of chaotic PID controlling PSO is more than other chaotic PSO algorithms on most of the test functions. Such illustrates that chaotic PID controlling PSO is required to learn from CPSO-3 and CPSO-2 and further refine the complex computational process so as to improve its efficiency.

### 3.5 Experimental discussion

In [6], CPSO-1 is considered to outperform other meta-heuristics such as PRS, MS, SA, TS, CSA and GA when solving complex optimization problems. Furthermore, in [11], CPSO-3 has better search ability than catfish PSO, SPSO and CenterPSO when searching for the global optima. Therefore, chaotic PID controlling PSO shows better search efficiency and quality, compared with these algorithms. The experimental results have proved the fact. The reason why chaotic PID controlling PSO yields better results for solving complex optimization problems is that the time varying PID controller, chaotic random parameters and chaotic local search for the global best position have effectively improved the evolutionary dynamics of particles and enhanced the particles’ local and global search exploration and exploitation abilities. However, these hybrid evolutionary strategies have to be further updated for high dimensional complex multimodal problems since the diversity of swarm becomes promptly deteriorated.

## 4 Application in parameter estimation of a nonlinear dynamic system

In this part, we conduct a detailed application to identifying the parameters of a nonlinear dynamic system. The application includes the description of the nonlinear dynamic system and experimental setup, parameter estimation and experimental results as well as model validation.

### 4.1 Description of the nonlinear dynamic system and experimental setup

In order to clarify the effectiveness and efficiency of chaotic PID controlling PSO, we apply chaotic PID controlling PSO, CPSO-3, GA and PSO to identifying the parameters of a nonlinear dynamic system. The nonlinear dynamic system is described as follow:

$$G\left(s\right)=\frac{K\cdot e^{-T_{3}s}}{\left(T_{1}s+1\right)\left(T_{2}s+1\right)}.$$

**Fig 5:**
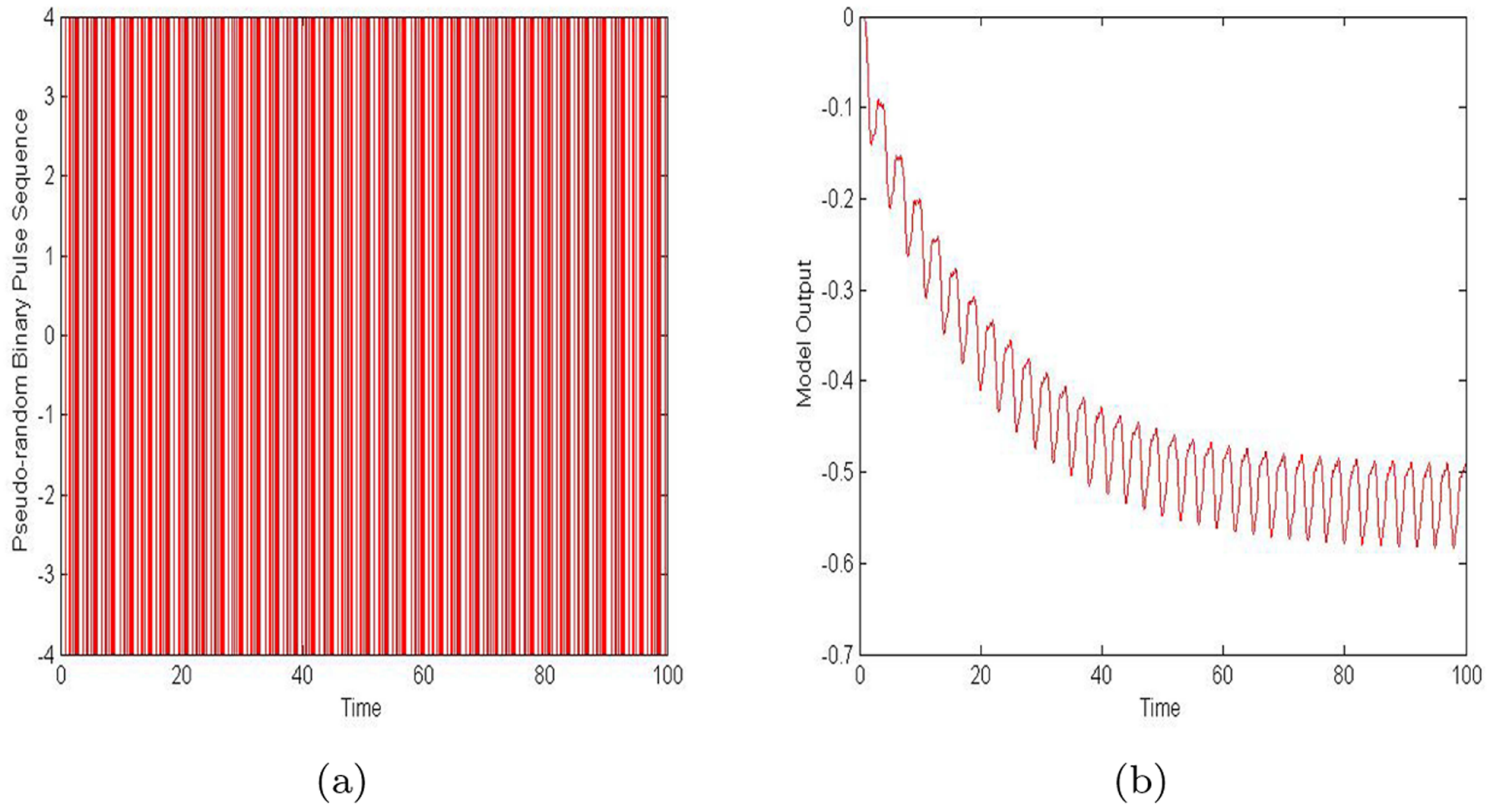
The input signal and its output are shown in the course of the estimation procedure. (a) The pseudo-random binary sequence. (b) The testing samples.

In the nonlinear dynamic model, the identified parameters *K*, *T*_1_, *T*_2_ *and* *T*_3_ are limited around the ranges [0, 30], [0, 10], [0, 30] and [0, 1], respectively. For the sake of the comparison to the experimental results, these real parameters are presented, namely *K* = 2, *T*_1_ = 1, *T*_2_ = 20 *and* *T*_3_ = 0.8. During the course of the parameter estimation, the identification criterion function is generally defined below

$$\begin{array}{c} E=\sum_{i=1}^{N}\frac{1}{2}{\left(y_{i}-{\hat{y}}_{i}\right)}^{T}\left(y_{i}-{\hat{y}}_{i}\right) \end{array}$$ (19),

where *N* is the number of testing samples, *y*_*i*_ is the output value of the *ith* testing sample, and ${\hat{y}}_{i}$ is the estimated prediction value of the *ith* testing sample. These testing samples are acquired when a pseudo-random binary sequence is regarded as the input signal. The pseudo-random binary sequence and the testing samples are shown in Fig 5 below.

For CPSO-3, GA, PSO and chaotic PID controlling PSO, the population size *PN* is set at 80, and the maximum generation number *MaxT* is set at 50. The settings of important parameters for GA and PSO are summarized in Table 9.

**Table 9:** Parameters settings for the involved optimization algorithms.

Name	Inertia Weight	Acceleration Coefficients and Others
GA		$CP=0.80\;\;\;MP=0.10-\frac{0.01\cdot t}{MaxT}$
PSO	$w_{pso}\left(t\right)=0.9-\frac{0.5\cdot t}{MaxT}$	*c*_1_(*t*) = *c*_2_(*t*) = 1.49445

### 4.2 Parameter estimation and experimental results

We wish to test CPSO-3, GA, PSO and chaotic PID controlling PSO on the above specific criterion fitness function with the 4-D parameters *K*, *T*_1_, *T*_2_ *and* *T*_3_ so as to estimate these parameters. To ensure the validation and accuracy of the experimental measurements, all evolutionary optimization algorithms are run 10 times on the fitness function and their final results are counted in the mean best fitness. The mean values, standard deviation of the results, and the best values are presented in Table 10 below. And in order to determine whether the results obtained by chaotic PID controlling PSO are statistically different from the results generated by other evolutionary optimization algorithms, the nonparametric Wilcoxon rank sum tests are conducted between the chaotic PID controlling PSO’s result and the result achieved by other evolutionary optimization algorithms for the fitness function.

**Table 10:** Results of diverse evolutionary optimization algorithms for the 4-D identification problem.

Results		CPSO-3	GA	PSO	CP_ID_SO	h_t-tests
*fitness*	Mean	9.6144e-007	0.0934	7.76500e-005	**1.3474e-011**	1
	Std. Dev	7.2614e-007	0.1015	1.000832e-004	**1.6508e-011**	
	Best	1.6929e-007	3.6800e-004	2.9727e-006	**3.1987e-014**	
*K*	Mean	1.9999	1.9817	1.9997	**2.0000**	
	Std. Dev	0.0002	0.1012	0.0010	**0**	
	Best	2.0000	1.9949	1.9999	**2.0000**	
*T*_1_	Mean	1.0002	1.0489	1.0021	**1.0000**	
	Std. Dev	0.0011	0.1311	0.0043	**0**	
	Best	0.9997	1.0043	0.9983	**1.0000**	
*T*_2_	Mean	19.9986	19.7638	19.9842	**20.0000**	
	Std. Dev	0.0079	3.3626	0.0244	**0**	
	Best	19.9991	19.8607	19.9960	**20.0000**	
*T*_3_	Mean	0.7998	0.7858	0.7975	**0.8000**	
	Std. Dev	0.0004	0.0079	0.0036	**0**	
	Best	0.8002	0.7941	0.7997	**0.8000**	

**Fig 6:**
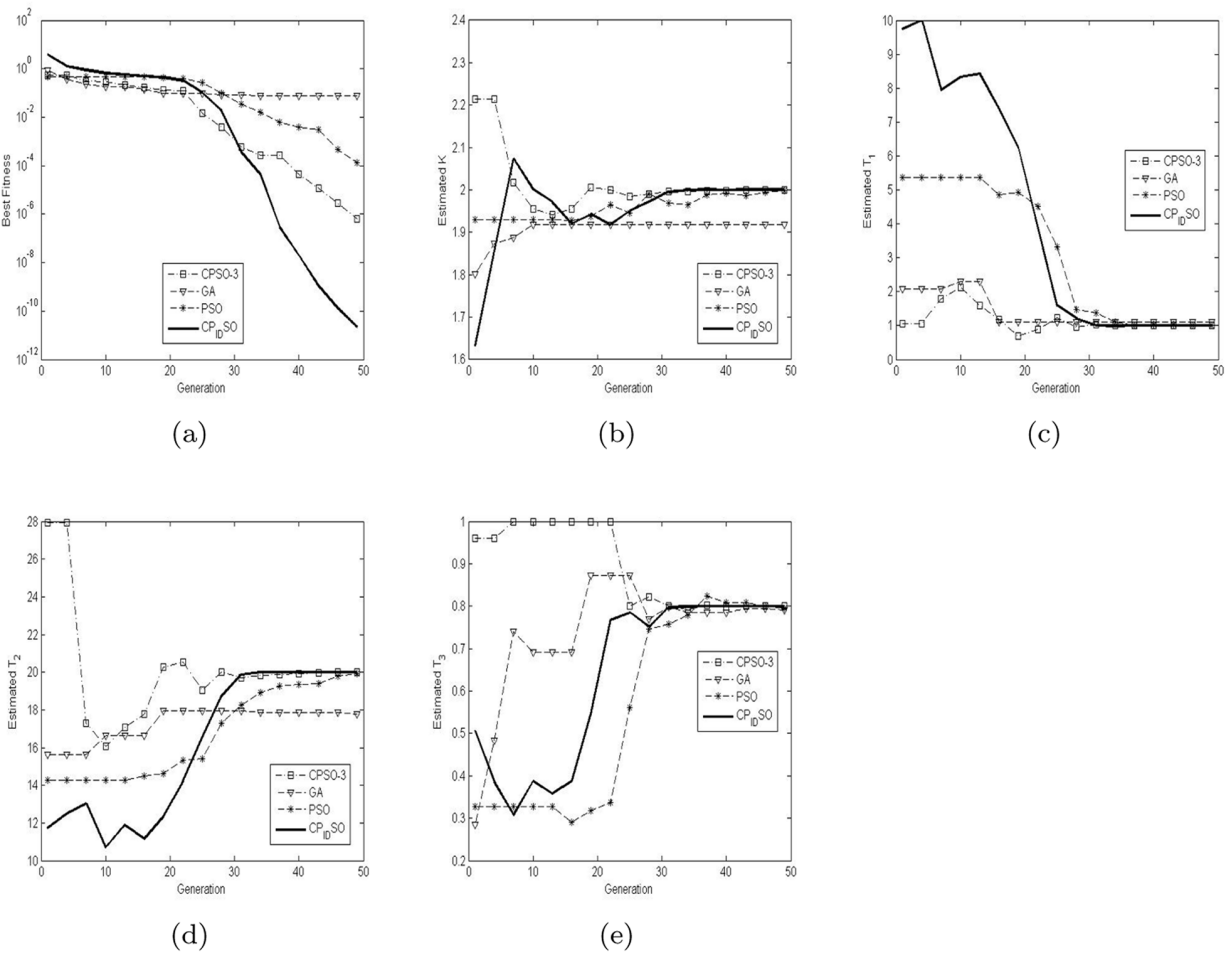
The median convergence and identification characteristics of diverse evolutionary optimization algorithms for 4-D identification problem above. (a) The median convergence characteristics of diverse evolutionary optimization algorithms. (b) The median identification characteristics of diverse evolutionary optimization algorithms for *K*. (c) The median identification characteristics of diverse evolutionary optimization algorithms for *T*_1_. (d) The median identification characteristics of diverse evolutionary optimization algorithms for *T*_2_. (e) The median identification characteristics of diverse evolutionary optimization algorithms for *T*_3_.

Table 10 presents the means and variances of the 10 runs of the four evolutionary optimization algorithms on the above specific criterion fitness function with its dimension 4. The best results among the evolutionary optimization algorithms are shown in bold in Table 10. Fig 6 presents the convergence and identification characteristics in terms of the best fitness value and parameters of the median run of diverse evolutionary optimization algorithms for the above specific criterion fitness function with its dimension 4. The results of the proposed chaotic PID controlling PSO are depicted by solid lines in Fig 6. Table 11 presents the average computational cost time (seconds) of diverse chaotic PSO algorithms for the 4-D identification problem.

**Table 11:** The average computational cost time (seconds) of diverse chaotic PSO algorithms for the 4-D identification problem.

Dimensionality	CPSO-3	GA	SPSO	CP_ID_SO
4	62.3166	23.7082	18.9603	73.9166

From the results in Table 10, we clearly notice that CPSO-3, PSO and chaotic PID controlling PSO outperform GA in the course of identifying the parameters *K*, *T*_1_, *T*_2_ *and* *T*_3_. In addition, chaotic PID controlling PSO performs best for all the parameter estimation whilst CPSO-3 achieves better estimated results than PSO. From the graphs in Fig 6, one may observe that the fact that the mean fitness of GA is the worst one of all is evident, which reveals GA’s inferiority to other three evolutionary algorithms for the whole parameter identification. On the other hand, it is worth noting that compared to PSO, chaotic PID controlling PSO has improved a lot in spite of more computational time consumption.

### 4.3 Model validation

**Fig 7:**
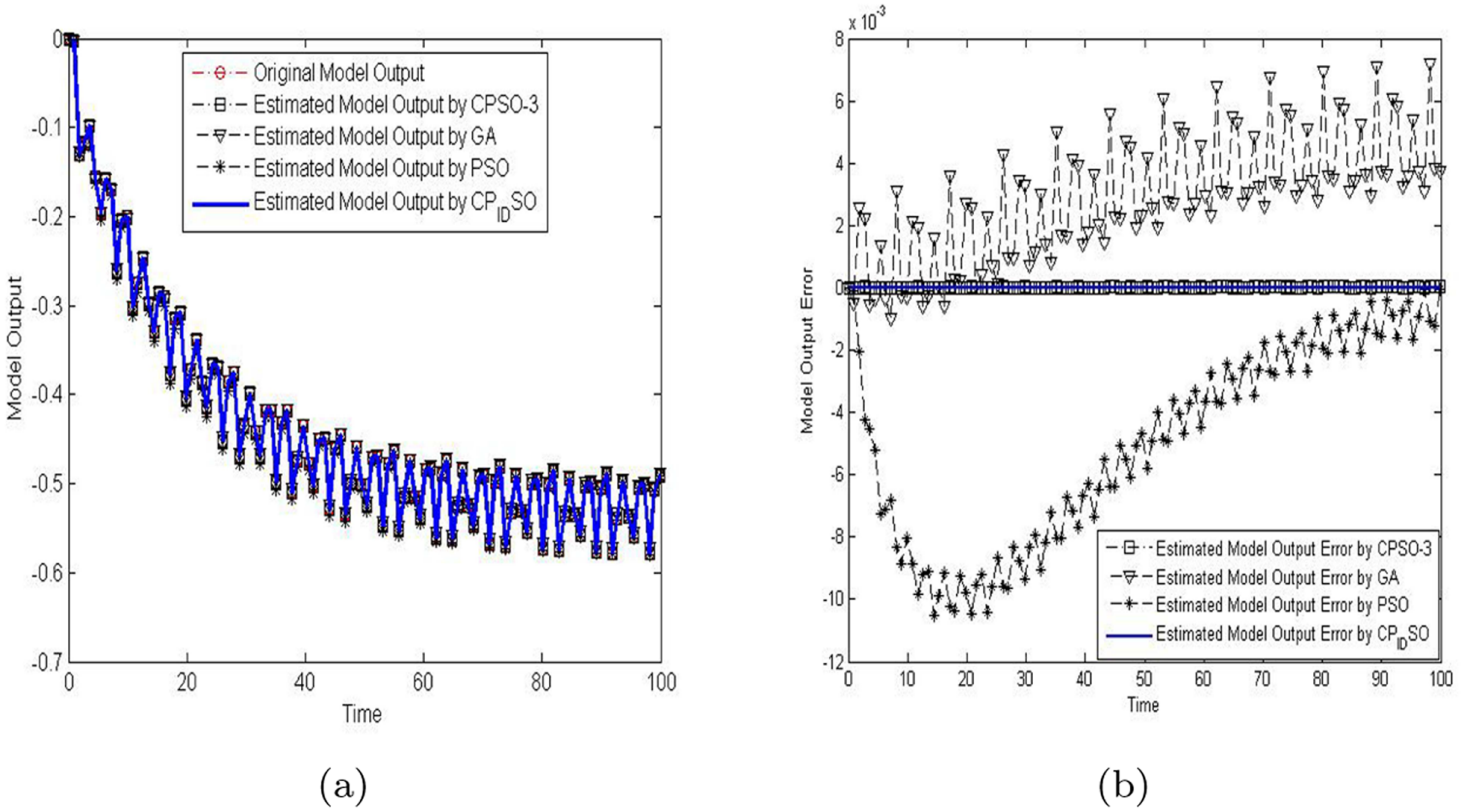
The output results and their errors of diverse evolutionary optimization algorithms for the 4-D identification problem are shown. (a) The output of diverse evolutionary optimization algorithms. (b) The output errors of the nonlinear dynamic system of diverse evolutionary optimization algorithms.

To verify the estimation results of the four evolutionary algorithms, their estimated parameters were used to the dynamic computation of the above nonlinear system. The concrete output results of the verification experiments are presented in Fig 7 and Table 12. Fig 7 presents the output results and their errors of diverse evolutionary optimization algorithms for the 4-D identification problem. Table 12 presents absolute accumulated errors by diverse evolutionary optimization algorithms for the 4-D identification problem.

**Table 12:** The absolute accumulated errors and parameter values of diverse evolutionary optimization algorithms for the 4-D identification problem.

Result	CPSO-3	GA	PSO	CP_ID_SO
*error*	2.6306e-007	0.0063	1.9272e-005	**0**
*K*	1.9999	1.9817	1.9997	**2.0000**
*T*_1_	1.0002	1.0489	1.0021	**1.0000**
*T*_2_	19.9986	19.7638	19.9842	**20.0000**
*T*_3_	0.7998	0.7858	0.7975	**0.8000**

As seen in Fig 7, there exist different absolute errors among these estimated output results. One may find that the absolute errors by chaotic PID controlling PSO and CPSO-3 are smaller while the ones by GA are biggest. In addition, PSO produces more accurate estimation results than GA. As given in Table 12, it is obvious that chaotic PID controlling PSO yields the best estimation results while GA performs comparatively worse. The estimation results by PSO are worse than those by CPSO-3, but are better than those by GA. Despite of these, all the evolutionary algorithms can be utilized to estimate the parameters of nonlinear dynamic systems.

## 5 Conclusions and future work

We present a chaotic PID controlling PSO variant, where we attempt to use the combination of a PID controller, chaotic logistic dynamics and hierarchical inertia weight to improve the performance of SPSO. Chaotic PID controlling PSO together with with other several chaotic PSO algorithms, is conducted on some multimodal functions. Successively, it is also used in the parameter identification of a given nonlinear dynamic system. The experimental results indicate chaotic PID controlling PSO enhances the diversity of swarm, and has better convergence efficiency, compared with other several chaotic PSO algorithms and meta-heuristics. Furthermore, chaotic PID controlling PSO also outperforms chaotic catfish PSO, GA and PSO for the parameter identification of the nonlinear dynamic system.

Future work will further the performances of hybrid evolutionary strategies of PID controllers, hierarchical inertia weight and chaotic dynamics for high dimensional complex multimodal problems. Besides, the refining of the multifarious computation is needed. Moreover, we will apply the proposed approach to other practical engineering applications.
